# Characterization of Virulence Properties of *Aeromonas veronii* Isolated from Diseased Gibel Carp (*Carassius* *gibelio*)

**DOI:** 10.3390/ijms17040496

**Published:** 2016-04-01

**Authors:** Jingjing Sun, Xiaojun Zhang, Xiaojian Gao, Qun Jiang, Yi Wen, Li Lin

**Affiliations:** 1 College of Animal Science and Technology, Yangzhou University, Yangzhou 225009, China; y0741810@126.com (J.S.); gaoxj336@163.com (X.G.); jiangqun1013@163.com (Q.J.); 2 College of Ocean, Huaihai Institute of Technology, Lianyungang 222005, China; 3 Bren School of Environmental Science and Management, University of California, Santa Barbara, CA 93106, USA; ywen@umail.ucsb.edu; 4 Department of Aquatic Animal Medicine, College of Fisheries, Huazhong Agricultural University, Wuhan 430070, China

**Keywords:** *Aeromonas veronii*, *Carassius gibelio*, virulence gene, proteinase, antibiotic resistance

## Abstract

*Aeromonas veronii* is a kind of opportunistic pathogen to fish and humans, significantly impending aquaculture production. Recently, we isolated two *A. veronii* strains, named GYC1 and GYC2, from diseased Gibel carp (*Carassius* *gibelio*) in China. Based on *gyr*B (DNA gyrase B subunit) genes of GYC1 and GYC2, the constructed phylogenetic tree showed that the two strains were clustered with *A. veronii*. Sixteen virulence genes related to the pathogenicity of *Aeromonas* spp. were subjected to PCR assay. The genes of *omp*AI, *omp*AII, *laf*A, *act*, *aer*, *fla*, *gca*T and *acg* were detected in the two strains, while genes of *hly*, *ahp*, *lip*, *ast* and *alt* were not detected. Additionally, genes *epr*CAI, *ela* and *exu* were only detected in the strain GYC1. Furthermore, the results of extracellular enzyme analysis revealed that the two isolates can produce hemolysin, caseinase, esterase, amylase and lecithinase, which were closely related to the pathogenicity of the two strains. However, the results showed that there was no gelatinase activity in either strain. According to the antibiotic resistant assay, the two strains were sensitive to cephalosporins and aminoglycosides, while they were resistant to penicillins and quinolones. Through this study, the virulence characteristics, including virulence genes and extracellular enzymes, the pathogenicity of *A. veronii* was clarified, enhancing the understanding about this pathogenic bacterium and providing the theoretical basis in disease control.

## 1. Introduction

The species from the genus *Aeromonas* of family Aeromonadaceae are considered to be emerging pathogens and clinical attention surrounding them has risen in aquaculture as well as in avian and human health. *A. hydrophila*, *A. veronii*, *A. sobria* and *A. caviae* are the major pathogenic bacteria for Aeromoniasis [1]. Currently there are 25 species in the genus *Aeromonas* [2]. Among them, *A. veronii*, as an opportunistic pathogen, seems to have the broadest host range in virulence which has been reported to cause wound infections, diarrhea and septicemia in humans [3,4]. Additionally, *A. veronii* has been reported to be a digestive tract symbiont of zebra fish and medicinal leech [2,5,6,7,8,9].

It is widely known that the pathogenesis of *Aeromonas* infections is due to multiple virulence-related factors including biologically active substances, adhesion organs and extracellular factors such as enzymes and toxins [3,10]. These different toxins and enzymes include lipase (*lip*), serine protease (*ser*), temperature-sensitive protease (*epr*CAI), aerolysin (*aer*), *omp*AI, *omp*AII, collagenase (*acg*), elastase (*ela*), cytotonic enterotoxins (*act*, *ast*, *alt*), and glycerophospholipids such as cholesterol acyltransferase (*gca*T), elastase (*ahy*B), DNases (*exu*), *etc.* These virulence-encoded genes have been widely used in determining the potential pathogenicity of *Aeromonas* species [11,12]. The extracellular enzymes which can cause host cell damage and degeneration would facilitate the pathogen in invading the host and establishing infection [13]. The pathogenicity of *Aeromonas* species is due to the combination of multiple virulence factors. Nowadays, it is hard to tell or define their role in the disease-causing process. That is to say, continuously surveying the presence of several accepted virulence-related factors in clinical *Aeromonas* isolates is essential to understanding the pathogenesis and epidemiology of *Aeromonas* [14,15,16].

*Aeromonas* species are ubiquitous Gram-negative bacilli found in aquatic environments. The involvement of *A. veronii* can cause the infected fish, with internal and surface bleeding accompanied by a high mortality rate. Gibel carp (*Carassius* *gibelio*), introduced into aquaculture as an important commercial fish species in China in the late 20th century, has been bred with a rapid growth rate. To reduce the influence of bacteria in this commercially important species is meaningful. In this study, we isolated two dominant *A. veronii* strains from diseased Gibel carp. Furthermore, the phylogenetic tree, virulence genes, extracellular enzymes and antibiotic sensitivity were characterized. The results will clarify molecular and phenotypic characteristics and, especially, the virulent traits of *A. veronii* to provide the theoretical basis in disease control.

## 2. Results

### 2.1. Diseased Fish and Gross Examination

Diseased Gibel carp exhibited dirty, swelling and congestion of the gill filament, and bleeding of jaw and operculum. Internally, diseased fish exhibited a distended gallbladder with some intestine and abdominal cavity effusion.

### 2.2. Physiological and Biochemical Characteristics

The results of the physiological and biochemical characteristics of the isolates are listed in Table 1. Additionally, the representative reactions such as H_2_S production, lactose, and the Voges–Proskauer (V–P) test and oxidation/fermentation (O–F) test were consistent with the data of *A. veronii* from *Bergey’s Manual of Determinative Bacteriology* [17], which were also listed in Table 1.

### 2.3. Molecular Characterization

**Figure 1:**
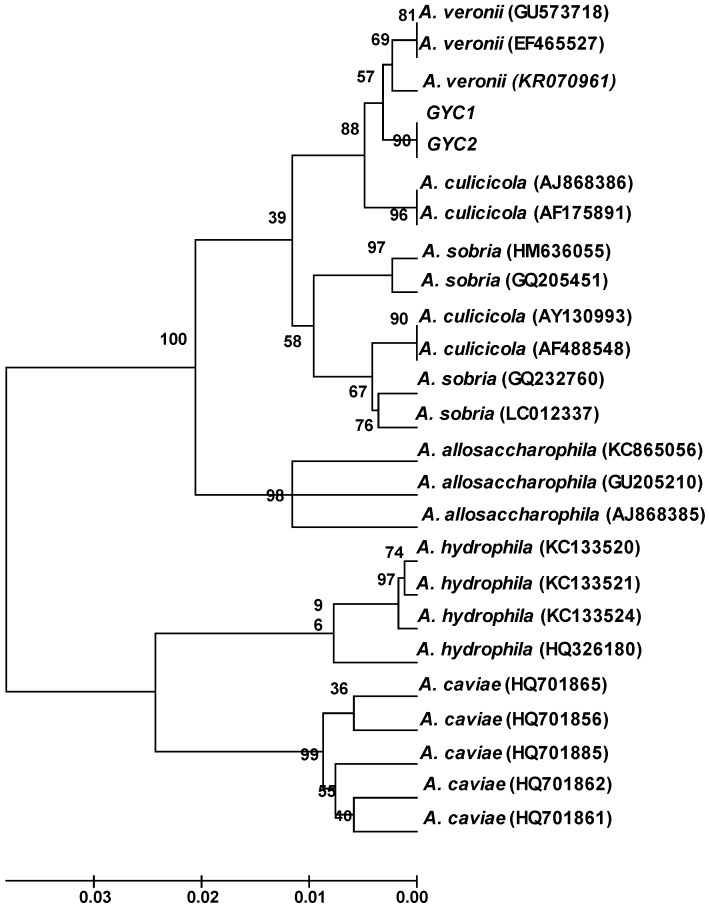
Phylogenetic tree based on the partial *gyr*B gene sequences (numbers in tree are bootstrap values).

In order to get the phylogenetic information about the studied strains GYC1 and GYC2, we determined the DNA sequences of *gyr*B genes from the isolates. Meanwhile, *A. veronii*, *A. culicicola*, *A. sobria*, *A. allosaccharophila*, *A. hydrophila* and *A. caviae* were chosen to build the phylogenetic tree. The results showed that GYC1 and GYC2 have been related to *A. veronii*, which is supported by a high bootstrap value (Figure 1). The phylogenetic tree was constructed according to Zhang *et al.* [18]

### 2.4. Experimental Infection

The fish experimentally infected with GYC1 and GYC2 showed identical symptoms as observed in the diseased Gibel carp during the disease outbreak in Ganyu County. Furthermore, *A. veronii* was re-isolated from the experimental infected fish, as confirmed by colonial morphology observation and the results of physiological and biochemical characteristics analysis. All fish injected with the isolates died from the fourth to seventh day post-injection. There were no clinical symptoms or death in the control groups. These results demonstrated that the isolated *A. veronii* was the pathogen of the Gibel carp.

### 2.5. Determination of Extracellular Enzymes and Hemolysin Activities

It is important to differentiate pathogenic and non-pathogenic *A. veronii* strains as it is only the pathogenic strains that can cause serious diseases in fish. In this study, we investigated hemolysin activity as well as lytic enzymes of the isolated strains, including caseinase, esterase, amylase, lecithinase and gelatinase, which are closely correlated with the bacterial virulence properties [19]. The results of extracellular enzymes and hemolysin activities were given in Table 2. Both strains have hemolysin activity and could produce caseinase, esterase, amylase and lecithinase. However, no gelatinase activity was detected.

### 2.6. Detection of Virulence Genes

**Figure 2:**
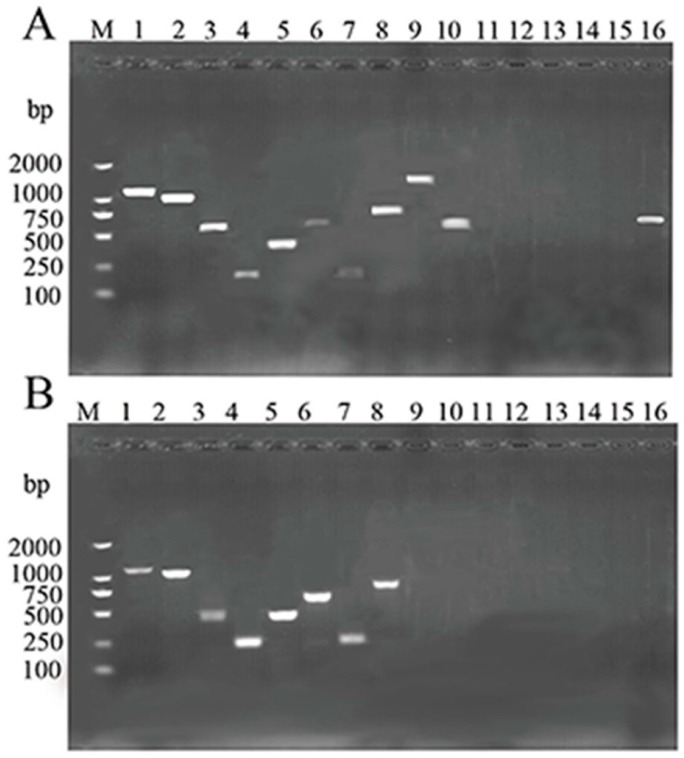
Agarose gel electrophoresis of 1% agarose of the amplification products of isolates GYC1 and GYC2 isolated from Gibel carp. M, Trans2K DNA Marker; lane 1, *omp*AI; lane 2, *omp*AII; lane 3, *laf*A; lane 4, *act*; lane 5, *aer*; lane 6, *fla*; lane 7, *gca*T; lane 8, *acg*; lane 9, *epr*CAI; lane 10, *ela*; lane 11, *hly*; lane 12, *ahp*; lane 13, *lip*; lane 14, *ast*; lane 15, *alt*; lane 16, *exu*; (**A**) GYC1; (**B**) GYC2.

There would be either one or a couple of virulence-related genes in *A. veronii* isolates which participate in the development of diseases in fish. In this study, 16 virulence genes (*omp*AI, *omp*AII, *laf*A, *act*, *aer*, *fla*, *gca*T, *acg*, *epr*CAI, *ela*, *hly*, *ahp*, *lip*, *ast*, *alt*, *exu*) were screened by PCR assay. The PCR profiles of the amplified virulence genes from the two *A. veronii* strains are presented in Figure 2. The genes *omp*AI, *omp*AII, *laf*A, *act*, *aer*, *fla*, *gca*T and *acg* were detected in both the two strains, while the genes *hly*, *ahp*, *lip*, *ast* and *alt* were absent. However, the genes *epr*CAI, *ela* and *exu* were detected in the strain GYC1, but were absent in GYC2.

### 2.7. Antibiotic Sensitivity

The antibiotic resistance patterns of the *A. veronii*, valued by the size of the inhibition zones around each disc, showed that the two strains were sensitive to cephradine, cefoperazone, cefotaxime, cefuroxime, cefoxitin, cefepime, clarithromycin, gentamycin, tobramycin, streptomycin, streptomycin and chloramphenicol. However, they were resistant to oxacillin, penicillin G, cefazolin, levofloxacin, ofloxacin, ciprofloxacin, norfloxacin (Table 3).

## 3. Discussion

The genus *Aeromonas* comprises a group of Gram-negative, facultative anaerobic bacteria that are opportunistic pathogens to aquatic and terrestrial animals, including humans [20]. In this report, we isolated two *A. veronii* strains (GYC1, GYC2) which have caused serious diseases of Gibel carp cultured in Jiangsu Province, China. The 16S rRNA gene sequence is a commonly used index in the construction of the phylogenetic tree of bacterial genera. Nevertheless, difficulties often arise when using this technique for species identification within *Aeromonas* spp. due to its smaller discriminatory power [21]. Compared to 16S rRNA, the gene of *gyr*B is more suitable for distinguishing *Aeromonas* at the species level for its higher expression level. Therefore, in this report, the phylogenetic tree was built on the basis of the *gyr*B gene of the bacteria, and the results showed that GYC1 and GYC2 clearly have a high relatedness to *A. veronii*, supported by a high bootstrap value.

The determination of extracellular enzymes and hemolysin is a direct way to manifest the virulence of a bacterium. The results indicated that both strains produced caseinase, esterase, and amylase lecithinase, and they contained hemolysin activity, supporting the strong virulence of the two isolates in Gibel carp cultured in China. Furthermore, virulence factor genes are also good markers for identifying the pathogenicity of a given microorganism. In this study, we studied 16 virulence genes related to *Aeromonas*. The results showed that the virulence genes *epr*CAI, *ela* and *exu* were only detected in GYC1, indicating that even the pathogenic strains may vary in their degree of virulence. On the other hand, the *omp*AI, *omp*AII, *laf*A, *act*, *aer*, *fla*, *gca*T and *acg* genes were present in both strains. These genes have been shown to be closely related with the pathogenicity of *Aeromonas.* Namba *et al.* [22] reported that *omp*A was an adhesion factor of *A. veronii* which was isolated from the carp intestinal tract. Gao *et al.* [23] found that mice that received *omp*A-*hly* antigen-loaded poly(lactic-*co*-glycolic) acid (PLGA) microspheres by intraperitoneal or intragastric administration mounted a strong and sustained IgG response. Lateral (laf) flagella are important in certain *Aeromonas* species for the adherence process and biofilm formation [24,25]. Kirov *et al.* [25] showed that, in general, all species of mutant *Aeromonad* defective in genes fla demonstrate a sharply decreased ability to form biofilms compared with the wild types. Additionally, *A. caviae* strains lacking lateral flagella resulted in a 60% decrease in adhesion to cells of the intestinal cell lines Henle 407 or Caco-2. In the research of Sen *et al.* [15], the swarming motility of tested strains corresponded with the presence of lafA in all *A. hydrophila*, *A. caviae*, and *A. veronii*. At the same time, only those strains that had one or more of the enterotoxins *fla*A, *fla*B, and either *fla*G or *laf*A, showed signs of being virulent. Han *et al.* [19] indicated the involvement of the collagenase gene in the pathogenesis of *A. veronii*. Adhesion and invasion abilities of the mutant strain on epithelioma papillosum of carp cells were only 56% of that of the wild-type strain, and the cytotoxicity was only 42%. It is noticeable that the genes *hly*, *ahp*, *lip*, *ast* and *alt* were absent in our two *A. veronii* strains. These results are consistent to some degree with Mohamed *et al.* [26], who found that in the 81 strains of *A. veronii* isolated from farm-raised catfish, none of the isolates contained the *ast* or *alt* gene. Based on our results, there may be an effective way to build an *A. veronii* detection method according to the mutual virulence genes, and there may even be an applicable way to construct a multivalent vaccine against the *A. veronii* infection.

The antibiotic sensitivity test demonstrated that both strains were sensitive to cephradine, cefoperazone, cefotaxime, cefuroxime, cefoxitin, cefepime, clarithromycin, gentamycin, tobramycin, streptomycin, streptomycin and chloramphenicol. Overall, the two strains were sensitive to cephalosporins and aminoglycosides. Antibiotics, no doubt, are an economical and effective option to fight against bacteria. However, the wide usage of antibiotics in clinical practice, veterinary medicine and agriculture has resulted in the release of large amounts of these pollutants to the environment [27]. Antibiotics, in addition to being chemical pollutants, exert a selective pressure retaining and spreading the various antibiotic resistance genes (ARG) among microbiota, which poses a risk to human health [28]. For example, the two isolated *A. veronii* strains already extended resistance to oxacillin, penicillin G, cefazolin, levofloxacin, ofloxacin, ciprofloxacin, and norfloxacin which were widely used in aquaculture. Instead of using antibiotics, the development of more effective strategies to fight bacterial infections is greatly expected in the future.

## 4. Materials and Methods

### 4.1. Diseased Fish and Gross Examination

The diseased Gibel carp were collected from Ganyu County, Jiangsu Province, China. The surfaces of each fish were examined for lesions and signs of physical damage. Post-mortem internal examinations were then conducted and wet mounts were taken from scrapes of the gills and skin and examined for the presence of protozoan and metazoan parasites.

### 4.2. Isolation of the Associated Bacteria

Abdominal cavities of diseased fish were opened up after surface sterilization with 70% ethanol. Samples were taken aseptically with scissors and tweezers from the liver, kidney and spleen tissues of freshly dead fish. The samples were streaked onto nutrient agar plate and incubated at 28 °C for 24 h. After incubation, bacterial colonies were selected based on the size, shape and color and purified before being stored at −80 °C in nutrient broth with 30% glycerol prior to identification.

### 4.3. Identification of the Isolates

#### 4.3.1. Physiological and Biochemical Characteristics

Physiological and biochemical examinations were carried out using standard plate and tube tests (Hangzhou Tianhe Microorganism Reagent Co., Ltd., Hangzhou, China) referring to *Bergey’s Manual of Systematic Bacteriology* [17], including the following tests: arabitol, mannose, maltose, H_2_S production, tartrate utilization, V–P test, xylose, mannitol, sucrose, galactosidase, aesculin, dulcitol, lactose, inositol, mushroom sugar, nitrate reduction, acetate, α-methyl-d-glucoside, galactose, sorbitol, O–F test, erythrite, l-rhamnose. The two strains were tested in twice and a third experiment would be carried out to eliminate the discrepancies when the results were different. The reactions were compared with the results of *A. veronii* from *Bergey’s Manual of Systematic Bacteriology*.

#### 4.3.2. Molecular Characterization

The chromosomal DNA from *A. veronii* was extracted using the EasyPure Genomic DNA Kit (Transgen Biotech, Beijing, China) in accordance with the manufacturer’s instructions. A partial 16S rRNA and *gyr*B genes sequence were amplified as described previously [18]. Briefly, universal PCR primers 27F (5′-AGAGTTTGATCMTGGCTCAG-3′) and 1492R (5′-TACGGMTACCTTGTTACGACTT-3′) were used for amplification of the 16S rRNA gene [29], while universal PCR primers UP1 (5′-GAAGTCATCATGACCGTTCTGCAYGCNGGNGGNAARTTYGA-3′) and UP2r (5′-AGCAGGGTACGGATGTGCGAGCCRTCNACRTCNGCRTCNGTCAT-3′) were used for amplification of the *gyr*B gene [30]. Polymerase chain reaction (PCR) amplification was performed in a total volume of 25 μL containing the appropriate reaction buffer and reagents: 2× EasyTaq PCR SuperMix (Transgen Biotech, Beijing, China) 12.5 μL, 1 μL (10 μM) forward and reverse primers, respectively, 9.5 μL ddH_2_O and 1 μL DNA as template. The PCR cycling protocol was as follows: a first denaturation at 95 °C for 5 min, followed by 30 cycles of denaturation at 95 °C for 1 min, annealing at 55 °C for 1 min and extension at 72 °C for 1 min. After a final extension at 72 °C for 10 min, the tubes were cooled to 4 °C. The amplification of 16S rRNA and *gyr*B genes and the construction of phylogenetic trees using MEGA4 were conducted according to Zhang *et al.* [18].

#### 4.3.3. Nucleotide Accession Numbers

The partial nucleotide sequences of 16S rRNA and gyrB locus of *A. veronii* strains were submitted to the GeneBank. The accession number of 16S rRNA and *gyr*B genes for GYC1 was KU543614 and KU543616, respectively, while the accession number of 16S rRNA and *gyr*B genes for GYC2 was KU543615 and KU543617, respectively.

### 4.4. Evaluation the Virulence of Isolates

#### 4.4.1. Experimental Infection

To confirm the pathogenicity of GYC1 and GYC2, experimental infections were conducted. Seventy healthy Gibel carp were purchased from fish farms located in Lianyungang City of Jiangsu Province, China, and have been maintained in still water supplemented with oxygen in 200 L aquaria at 20 °C for 10 days. Groups of 10 healthy Gibel carp (average 10–15 cm in length) were injected intraperitoneally with 0.1 mL live cells (containing 10^6^, 10^7^ or 10^8^ CFU·mL^−1^, respectively) suspended in saline per fish, while the other 10 fish were similarly injected with 0.1 mL saline and were used as negative controls. All mortalities of inoculated fish kept at 20 °C were recorded lasting 10 days. Dead and moribund fish were removed for pathological examination and external and internal signs of disease were recorded. Samples of the liver, spleen and kidney were streaked onto 2216E marine agar plates and blood agar plates for bacteriological examination. The surviving fish were euthanized in the end of the experiment.

#### 4.4.2. Determination of Extracellular Enzymes and Hemolysin Activities

The presence of extracellular enzymes and hemolysin of the isolates were determined according to the methods reported by Yang *et al.* [31]. Hemolytic activity of the bacteria were detected by streaking onto the rabbit blood nutrient agar plates and incubated at 28 °C for 24 h. The presence of a clear colorless zone surrounding the colonies indicated β-hemolytic activity. Likewise, incomplete transparent zone indicates α-hemolytic activity.

Casein hydrolysis, amylase, gelatinase, lipase activity and lecithin hydrolysis were tested on nutrient agar containing 10% skimmed milk, 1% starch, 8% gelatin, 1% Tween 80 and 1% egg yolk, respectively. The extracellular enzymes were assayed by streaking the cells onto the plates and incubating at 28 °C for 24 h. The presence of a transparent zone surrounding the colonies indicated the particular enzyme activity. All treatments were performed in triplicate.

#### 4.4.3. Detection of Virulence Genes

The two *A. veronii* strains were subjected to PCR assays to detect the 16 genes encoding the toxins, including *omp*AI, *omp*AII, lateral flagella (*laf*A), cytotonic enterotoxins (*act*, *ast*, *alt*), aerolysin (*aer*), the structural gene flagellin (*fla*), glycerophospholipid: cholesterol acyltransferase (*gca*T), collagenase (*acg*), temperature-sensitive protease (*epr*CAI), elastase (*ela*), hemolysin (*hly*), serine protease (*ahp*), lipase (*lip*), DNases (*exu*). Oligonucleotide primers, target genes, and amplicon sizes are shown in Table 4. The final concentrations in the PCR mixture were as follows: 2× EasyTaq PCR SuperMix 12.5, 1 μL (10 μM) forward and reverse primers, respectively, 9.5 μL ddH_2_O and 1 μL DNA as template. The thermocycling program was optimized as follows: a first denaturation at 95 °C for 5 min, then 30 cycles of denaturation at 95 °C for 1 min, annealing at 55 °C for 1 min and extension at 72 °C for 1 min. After a final extension at 72 °C for 10 min, the tubes were cooled to 4 °C. Aliquots from amplification reactions were analyzed by 1% agarose gel electrophoresis.

### 4.5. Antibiotic Sensitivity

The sensitivity of the two strains to 31 antibiotics listed in Table 3 was evaluated by disc diffusion method [36] on Muller-Hinton agar using commercial antibiotic discs (Hangzhou Tianhe Microorganism Reagent Co.). All the tests were performed in triplicate. Antibiotic sensitivity of the strains as resistant, intermediate or sensitive was measured by the size of the inhibition zones around each disc according to standards suggested by Hangzhou Tianhe Microorganism Reagent Co.

## 5. Conclusions

In this study, virulence properties of two bacterial strains (GYC1 and GYC2) from diseased Gibel carp in China were characterized. The physiological and biochemical characteristics of the isolates were consistant with the data of *A. veronii* from *Bergey’s Manual of Determinative Bacteriology* [17]. The constructed phylogenetic tree based on *gyr*B (DNA gyrase B subunit) genes of GYC1 and GYC2 showed that the two strains were clustered with *A. veronii*. Through PCR assay, 16 genes related to *Aeromonas* virulence were studied and the genes *omp*AI, *omp*AII, *laf*A, *act*, *aer*, *fla*, *gca*T and *acg* were found in both strains. Furthermore, the results of extracellular enzymes analysis revealed that the two isolates could produce hemolysin, caseinase, esterase, amylase and lecithinase while no gelatinase activity was detected in either strain. Experimental infection showed that the mortality of fish injected with the isolated bacteria was 100% within 10 days and the control group was zero, furtherly confirming the pathogenicity of the isolates. The antibiotic resistant assay revealed that the two strains were sensitive to cephalosporins and aminoglycosides, while they were resistant to penicillins and quinolones.

## Figures and Tables

**Table 1:** The represented physiological and biochemical characteristics of the isolates.

Items	Isolates		*A. veronii*
	GYC1	GYC2	
Arabitol	−	−	−
Mannose	+	+	+
Maltose	+	+	+
H_2_S production	−	−	−
Tartrate utilization	−	−	−
V–P test	+	+	+
Xylose	−	−	−
Mannitol	+	+	+
Sucrose	+	+	+
Galactosidase	+	+	[+]
Aesculin	+	+	+
Dulcitol	−	−	−
Lactose	−	−	−
Inositol	−	−	−
Mushroom sugar	+	+	+
Nitrate reduction	+	+	+
Acetate	−	−	[+]
α-methyl-d-glucoside	−	−	[+]
Galactose	+	+	+
Sorbitol	−	−	−
O–F test	F	F	F
Erythrite	−	−	−
l-Rhamnose	−	−	−

“+”, positive; “−”, negative; “F”, fermentative; ”[+]”, 76%–89% of the strains are positive; V–P test: Voges–Proskauer test; O–F test: oxidation/fermentation test.

**Table 2:** Production of extracellular enzymes and hemolysin activity of the isolated *A. veronii*.

Isolates	Caseinase	Esterase	Amylase	Lecithinase	Hemolysin	Gelatinase
GYC1	+	+	+	+	β-hemolysis	−
GYC2	+	+	+	+	β-hemolysis	−

“+”, positive; “"−”, negative.

**Table 3:** Antibiotic sensitivity of the two isolates.

Groups	Chemicals	Disc Content (μg)	Mean Inhibition Zone Diameter (mm)		Sensitivity ^a^	
			GYC1	GYC2	GYC1	GYC2
Penicillins	Oxacillin	1	0.0	0.0	R	R
	Piperacillin	100	26.0	0.0	S	R
	Penicillin G	10	15.0	0.0	R	R
	Ampicillin	10	13.0	0.0	I	R
Cephalosporins	Cephradine	30	25.0	24.0	S	S
	Cefoperazone	75	26.0	22.0	S	S
	Cefalotin	30	20.0	0.0	S	R
	Cefotaxime	30	27.0	25.0	S	S
	Cefuroxime	30	25.0	24.0	S	S
	Cefoxitin	30	20.0	20.0	S	S
	Cefepime	30	26.0	25.0	S	S
	Cefazolin	30	14.0	14.0	R	R
Macrolides	Midecamycin	30	16.0	14.0	I	I
	Clarithromycin	15	25.0	20.0	S	S
	Erythromycin	15	27.0	20.0	S	I
Quinolones	Levofloxacin	5	0.0	11.0	R	R
	Ofloxacin	5	9.0	10.0	R	R
	Ciprofloxacin	5	9.0	0.0	R	R
	Norfloxacin	10	0.0	10.0	R	R
Aminoglycosides	Gentamycin	10	19.0	16.0	S	S
	Tobramycin	10	18.0	20.0	S	S
	Streptomycin	10	22.0	15.0	S	S
	Streptomycin	30	20.0	18.0	S	S
	Amikacin	30	19.0	16.5	S	I
	Spectinomycin	100	27.0	0.0	S	R
Lincomycins	Clindamycin	2	15.0	12.5	I	R
Amphenicols	Chloramphenicol	30	22.0	22.0	S	S
Polymyxin	Polymyxin B	300	14.0	0.0	S	R
Nitrofuran	Macrodantin	300	18.0	16.0	S	I
Aztreonam	Aztreonam	30	26.0	10.0	S	R
Glycopeptides	Vancomycin	30	11.0	10.0	R	R

a: R, resistance; S, sensitive; I, intermediate.

**Table 4:** PCR primers, targets, and amplicon sizes used for this study.

Target Gene	Product Size (bp)	PCR Primers Sequence (5′-3′)	Reference
*omp*AI	1026	F: GACGATATCATGATGAAAATGGCTCTT	Wang Hui [32]
		R: GCGAAGCTTTTACTTCTGAACTTCTTG	
*omp*AII	1001	F: GCTGAATTCATGAAACTCAAAATGGCTC	Wang Hui [32]
		R: GCGAAGCTTTTACTGTTGTACTTGC	
*laf*A	550	F: GGTCTGCGCATCCAACTC	Merino *et al.* [33]
		R: GCTCCAGACGGTTGATG	
*act*	232	F: AGAAGGTGACCACCACCAAGAACA	Mohamed *et al.* [26]
		R: AACTGACATCGGCCTTGAACTC	
*aer*	431	F: CCTATGGCCTGAGCGAGAAG	Mohamed *et al.* [26]
		R: CCAGTTCCAGTCCCACCACT	
*fla*	608	F: TCCAACCGTYTGACCTC	Mohamed *et al.* [26]
		R: GMYTGGTTGCGRATGGT	
*gca*T	237	F: CTCCTGGAATCCCAAGTATCAG	Mohamed *et al.* [26]
		R: GGCAGGTTGAACAGCAGTATCT	
*acg*	761	F: AACAAGCACCCGTTAAGCCAC	Han *et al.* [19]
		R: ACGTAGTCGAGCCCCTTGAGG	
*epr*CAI	387	F: GCTCGACGCCCAGCTCACC	Ren *et al.* [34]
		R: GGCTCACCGCATTGGATTCG	
*ela*	513	F: ACACGGTCAAGGAGATCAAC	Sen and Rodgers [15]
		R: CGCTGGTGTTGGCCAGCAGG	
*hly*	597	F: GGCCGGTGGCCCGAAGATACGGG	Wong *et al.* [35]
		R: GGCGGCGCCGGACGAGACGGG	
*ahp*	911	F: ATTGGATCCCTGCCTA	Li *et al.* [11]
		R: GCTAAGCTTGCATCCG	
*lip*	382	F: ATCTTCTCCGACTGGTTCGG	Sen and Rodgers [15]
		R: CCGTGCCAGGACTGGGTCTT	
*ast*	331	F: TCTCCATGCTTCCCTTCCACT	Mohamed *et al.* [26]
		R: GTGTAGGGATTGAAGAAGCCG	
*alt*	442	F: TGACCCAGTCCTGGCACGGC	Yang and Fang [31]
		R: GGTGATCGATCACCACCAGC	
*exu*	323	F: AGACATG CACAACCTCTTCC	Yang and Fang [31]
		R: GATTGGTATTGCCTTGCAAG	
